# ADAM17 Mediates Hypoxia-Induced Keratinocyte Migration via the p38/MAPK Pathway

**DOI:** 10.1155/2021/8328216

**Published:** 2021-10-28

**Authors:** Guoqin Zhu, Jie Liu, Yuan Wang, Naixin Jia, Weiyi Wang, Junhui Zhang, Yi Liang, Hao Tian, Jiaping Zhang

**Affiliations:** ^1^Key Laboratory of Freshwater Fish Reproduction and Development, Ministry of Education, Laboratory of Molecular Developmental Biology, School of Life Sciences, Southwest University, Beibei, 400715 Chongqing, China; ^2^Department of Plastic and Aesthetic Surgery, State Key Laboratory of Trauma, Burns and Combined Injury, Southwest Hospital, The Third Military Medical University (Army Medical University), Chongqing 400038, China

## Abstract

Although hypoxia has been shown to promote keratinocyte migration and reepithelialization, the underlying molecular mechanisms remain largely unknown. ADAM17, a member of the metalloproteinase superfamily, has been implicated in a variety of cellular behaviors such as proliferation, adhesion, and migration. ADAM17 is known to promote cancer cell migration under hypoxia, and whether or how ADAM17 plays a role in hypoxia-induced keratinocyte migration has not been identified. Here, we found that ADAM17 expression and activity were significantly promoted in keratinocytes under hypoxic condition, inhibition of ADAM17 by TAPI-2, or silencing of ADAM17 using small interfering RNA which suppressed the hypoxia-induced migration of keratinocytes significantly, indicating a pivotal role for ADAM17 in keratinocyte migration. Further, we showed that p38/MAPK was activated by hypoxia. SB203580, an inhibitor of p38/MAPK, significantly attenuated the upregulation of ADAM17 as well as the migration of keratinocytes induced by hypoxia. Activation of p38/MAPK by MKK6 (Glu) overexpression, however, had adverse effects. Taken together, our study demonstrated that hypoxia-induced keratinocyte migration requires the p38/MAPK-ADAM17 signal axis, which sheds new light on the regulatory mechanisms of keratinocyte migration. Our study might also help in developing therapeutic strategies to facilitate wound healing in vivo, where cells are migrated in a hypoxic microenvironment.

## 1. Introduction

Wound healing, an important physiological process that restores the integrity of skin after trauma, consists of a sequence of well-characterized stages governed by sequential and overlapping phases, in which reepithelialization is the key stage depending upon keratinocyte migration from the wound margins [1]. Hypoxia, or a low oxygen concentration, is a microenvironmental hallmark of most solid cancers and wound healing. When the skin is injured, hypoxia occurs due to vascular disruption, vasoconstriction, and increased oxygen consumption [2]. Initially, evidence has identified hypoxia as an inherent impediment to cancer therapy due to its contributions to chemoresistance, angiogenesis, and invasiveness [3–5]. Hypoxia was also found to stimulate angiogenesis, granulated tissue formation, and wound repair in diabetic mice [6]. Moreover, relative hypoxia has been shown to promote keratinocyte migration and reepithelialization [7–9]. Although considerable research has explored the mechanisms by which hypoxia controls keratinocyte migration during wound healing, the underlying molecular mechanism remains largely unknown.

In response to hypoxia, such as hypoxia-inducible factor (HIF), nuclear factor (NF)-*κ*B, and specificity protein 1(Sp1), in which gene expression is involved in the transcription factors mentioned [10]. Involvement of transcription factors shown in the downstream signaling pathway, a disintegrin and metalloproteinase (ADAM) family, is one of the most studied [11]. ADAMs are multidomain transmembrane proteins containing a disintegrin-like and metalloprotease domain. ADAM17, also called tumor necrosis factor-alpha-converting enzyme (TACE), is a prominent member of the ADAM family [12]. It has been implicated in a variety of cellular and physiological processes, including cell proliferation [13], adhesion [14], and migration [15]. Results have shown that ADAM17 mRNA is significantly increased in migrating keratinocytes during wound healing [16]; in addition, blocking ADAM17 maturation inhibits keratinocyte migration [17] and hypoxia upregulates ADAM17 expression and promotes cell migration [18]. These studies indicate that ADAM17 plays a pivotal role in keratinocyte migration.

Importantly, our previous research is consistent with others that show the role of p38/MAPK signaling in hypoxia-induced keratinocyte migration [19, 20]. P38/MAPK is responsible for various cellular events under stresses such as cell survival, cell proliferation, cell cycle progression, and cell migration. A number of studies have revealed that ADAM17 is activated by MAPKs, including the p38/MAPK signaling in both epithelial cells and cancer cells [21, 22]. However, it is not clear whether there is a role for p38/MAPK in ADAM17-regulated keratinocyte migration under hypoxia. In this study, we have demonstrated for the first time that ADAM17 mediates keratinocyte migration under hypoxia through a p38/MAPK-dependent pathway. Our results showed that ADAM17 expression and activity are upregulated in hypoxic keratinocytes. Silencing or inhibition of ADAM17 suppressed the hypoxic keratinocyte migration. Meanwhile, we demonstrated that p38/MAPK played a critical role in ADAM17 expression as well as keratinocyte migration under hypoxia. Our study provided novel insights into the mechanisms of hypoxia-induced keratinocyte migration that requires the p38/MAPK-ADAM17 signal axis.

## 2. Materials and Methods

### 2.1. Cell Culture and Hypoxia Treatment

HaCaT cells were purchased from the cell bank of the Chinese Academy of Sciences in Beijing, China. Cells were cultured in RPMI 1640 medium (HyClone, USA) containing 10% fetal bovine serum (HyClone, USA), 100 U/ml penicillin (Invitrogen, USA), and 100 mg/ml streptomycin (Invitrogen, USA).The cells were then put in 37°C, 5% CO_2_ incubator, and 95% humidity.

Hypoxic conditions of 1% O_2_, 5% CO_2_, and 94% N_2_ were produced utilizing an oxygen control incubator (model: 3131; Thermo Scientific). The p38/MAPK inhibitor SB203580 (Beyotime) (5 *μ*mol/l) was added to these cultures and incubated at 37°C for 30 minutes before hypoxia treatment. In addition the ADAM17 inhibitor, TAPI-2 (40 *μ*M) was added to the cultures and incubated at 37°C for 12 hours before hypoxia treatment.

### 2.2. Western Blot Analysis

Phosphate-buffered saline (PBS) was used to wash cells, harvested in 70–200 *μ*l lysis buffer on ice, and homogenized. The lysates were sonicated for 4 seconds and centrifuged at 14000 rpm for 15 min at 4°C. Protein concentrations were observed by the BCA protein assay kit (Beyotime). Cell lysates containing 20 *μ*g of proteins were electrophoresed on 10% SDS-PAGE gels and transferred to a PVDF membrane (Millipore). The membranes were blocked in 5% bovine serum albumin (BSA) for 2 hours at room temperature. The following primary antibodies were used for detecting protein expression: anti-ADAM17 (1 : 1000, Abcam, USA), anti-p38-HRP (1 : 1000, Cell Signaling, USA), and anti-phospho-p38 at Thr180/Tyr182 (1 : 1000, Cell Signaling, USA). Horseradish peroxidase-conjugated IgG was used as a secondary antibody, and *β*-actin (1 : 4000, ProteinTech, USA) was used as a loading control. The immunocomplexes were imaged using an enhanced chemiluminescence detection kit (Amersham Pharmacia) and a ChemiDoc imaging system (Bio-Rad, USA). The images were quantified with the Quantity One 4.1 software (Bio-Rad, USA). Each experiment was repeated three times.

### 2.3. Transfection of HaCat Cells with ADAM17-siRNA

On-target siRNA specific to ADAM17 (siADAM17) and negative control siRNA (NC-siRNA) were obtained from GenePharma (China). The ADAM17 target sequences are shown in supplemental Table 1.

### 2.4. MKK6 (Glu) Recombinant Adenovirus Construction and Transduction

MKK6 (Glu) recombinant adenovirus vector and negative vector were produced by Shanghai GeneChem (China). Their transgenic expression in HaCaT cells was determined using Western blotting.

### 2.5. Cell Scratch Wound Healing Assay

The cell scratch wound assay is a straightforward and economical method to study keratinocyte migration collectively in vitro [23]. HaCaT cells uninfected or infected with recombinant adenoviruses or siADAM17 were grown to confluence in 24-well plates in serum-conditioned RPMI 1640. Scratch wounds were created in the confluent monolayers using a sterile p200 pipette tip, and different fields were captured under normoxic or hypoxic conditions. Four perpendicular semiopaque marks were placed across each scratch on the external surface of the well to standardize the quantitative analyses. After the adherent cells were washed three times, the wounded monolayers were cultured in RPMI 1640 medium. After incubation for the indicated hours, the repopulation of the wounded areas was observed under a Zeiss imaging system. Using the NIH ImageJ image processing program, the size of the denuded area was determined at each time point from the digital images.

### 2.6. Cell Motility Assay

HaCaT cells were seeded at a density of 0.5 × 10^5^/ml. Twenty-four-well plates received overnight incubation in 1 ml RPMI 1640 media which contained 10% FBS. The keratinocytes were pretreated and exposed to hypoxia. Time-lapse imaging was performed with a Zeiss imaging system (Carl Zeiss Meditec, Jena, Germany) that was equipped with a CO_2_- and temperature-controlled chamber and acquired the image each at 5 min. The time-lapse images were analyzed using NIH ImageJ software.

### 2.7. Cell Proliferation Assay

HaCaT cells were seeded at 2 × 10^3^/well in 96-well plates in RPMI 1640 medium supplemented with 10% FBS. Cell proliferation was determined by Cell Counting Kit-8 (CCK-8) (Dojindo Molecular Technologies, Rockville, MD, USA) assays according to the manufacturer's instructions. After inoculating the cell suspension in a 96-well plate, the plate was preincubated for the indicated time in a normoxia- or hypoxia-humidified incubator (at 37°C, 5% CO_2_). CCK-8 solution (10 *μ*l) was added to each well of the plate, and then, the plate was incubated for 1 hour. Finally, the absorbance was measured at 450 nm using a microplate reader (Thermo, USA). The experiment was repeated three times.

### 2.8. ADAM17 Activity Assay

For activity assay, a SensoLyte 520 TACE activity assay kit (AnaSpec Inc., USA) with the fluorogenic peptide OXLTM 520/5-FAM (ADAM17 cleavage site from TNF-*α*) was used. A total of 5 × 10^3^ HaCat cells per well were seeded in a 96-well plate. Twenty-four hours after seeding and then incubating with ADAM17 fluorescently labelled substrate for 30 minutes at 37°C, the relative fluorescence at 520 nm (excitation: 490 nm) was monitored as baseline activity. Afterwards, the cells were treated with an inhibitor and/or stimulant and the activity was monitored at 37°C for one hour. The activity of the unstimulated/stimulated cells was normalized to their baseline activity.

### 2.9. Substrate Shedding Assay (ELISA)

To assess the production of ADAM17 substrates, keratinocytes were incubated for different times with medium containing no exogenous EGFR ligands and then collected. To assess TNF-*α* and HB-EGF production, normal HaCaT cells were incubated in hypoxic and normoxic conditions for 0 hours, 12 hours, and 24 hours. The collected medium specimens were analyzed for the proteins of interest using the relevant DuoSet ELISA kits (R&D Systems, Abingdon, UK). In all cases, the data are presented as the mean ± SEM. Supernatant collection was performed in triplicate for each cell line, and the results from the control and vector control keratinocyte cell lines were separated for presentation. The results were analyzed by Student's *t*-test using GraphPad Prism (GraphPad, San Diego, CA, USA).

### 2.10. Statistical Analysis

All data obtained in the study are expressed as the mean ± SEM. The results were compared by a one-way analysis of variance, followed by the Student-Newman-Keuls *t*-test. Statistical was significance set at ^∗^*P* < 0.05, ^∗∗^*P* < 0.01, and ^∗∗∗^*P* < 0.001, and statistical analyses were performed using GraphPad Prism software. *P* < 0.05 was considered statistically significant.

## 3. Results

### 3.1. Hypoxia Promoted Keratinocyte Migration

**Figure 1:**
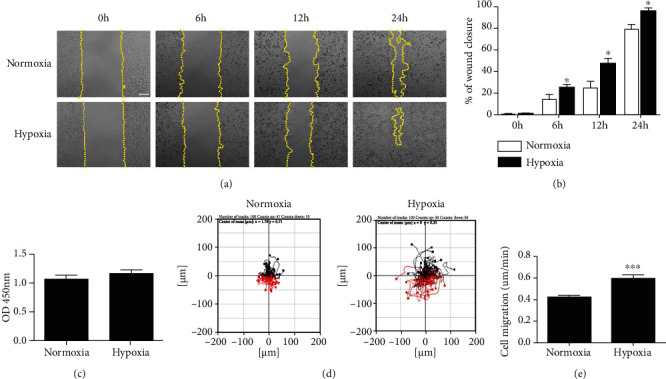
Hypoxia promoted keratinocyte migration. (a) HaCaT cells were subjected to scratch wounding with a micropipette tip (200 *μ*l) and then incubated under normoxic condition (21% O_2_) or hypoxic condition (1% O_2_) (0, 6, 12, and 24 hours). The cells were recorded by time-lapse video microscopy at different times. (b) Quantification analysis of wound healing assays using ImageJ software. (c) HaCaT cell proliferation was determined using a Cell Counting Kit-8 assay. (d) The effect of hypoxic (1% O_2_) treatment on cell motility trajectories in HaCaT cells, filmed for 12 hours using time-lapse video microscopy. (e) The statistical value of cell trajectory speed. Data were from at least 3 independent experiments and shown as the mean ± SEM. ^∗^*P* < 0.05 and ^∗∗∗^*P* < 0.001 vs the normoxia group. Bar = 100 *μ*m.

Hypoxia plays a crucial role in reepithelialization in wound healing. Nevertheless, hypoxia regulation of keratinocytes is associated with oxygen concentrations [24], and in the wound centre, the oxygen concentration is approximately 0–10 mmHg (0–1.3% O_2_) [25]. To assess whether hypoxia induces keratinocyte migration, we utilized cell scratch wound healing assay and cell motility assay. The results of cell scratch wounding assay showed that the migration of keratinocytes was enhanced after culture in hypoxic condition compared to that in normoxic condition (Figures 1(a) and 1(b)). After culturing for 6, 12, and 24 hours under hypoxic conditions, keratinocytes migrated into an area over 24.8 *μ*m^2^, 42.50 *μ*m^2^, and 95.63 *μ*m^2^, which occupied 25.36%, 43.93%, and 91.64% of the scratched area, respectively, while keratinocytes were under a normoxia area over only 14.01 *μ*m^2^, 24.94 *μ*m^2^, and 78.75 *μ*m^2^, which occupied 15.75%, 26.98%, and 80.92%, respectively (Figure 1(b)). No difference in cell proliferation was detected between the hypoxia group and the normoxia group (Figure 1(c)). Thus, cell proliferation can be excluded as a possible explanation for the differences in the migratory capacity of hypoxic keratinocytes. We further investigated the keratinocyte migration under hypoxia using cell motility assay. HaCaT cells were subjected to hypoxia or normoxia and were filmed for 12 hours using time-lapse video microscopy. As shown in Figures 1(d) and 1(e), after hypoxic pretreatment, the trajectory speed of keratinocytes was increased 1.4-fold, compared with normoxic condition (Supplemental Movie S1). These results suggest that hypoxia induced keratinocyte migration.

### 3.2. Hypoxia Increased ADAM17 Expression and Activity in Keratinocytes

**Figure 2:**
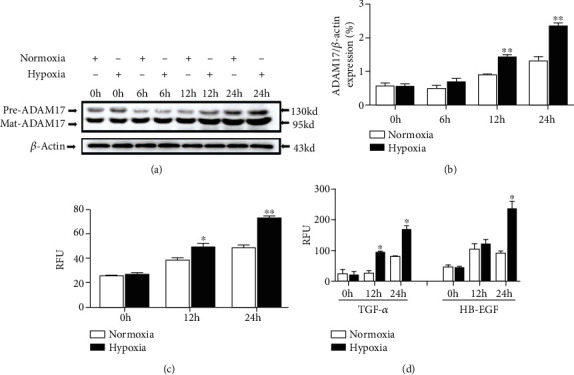
Hypoxia increased ADAM17 expression and activity in keratinocytes. (a) Western blotting was used to detect ADAM17 expression in HaCaT cells under hypoxic condition. (b) The graph represents the mean ± SEM (*n* = 3) of the relative ADAM17 precursor protein and mature protein-integrated Western blot signal. (c) A SensoLyte 520 TACE activity assay was performed to determine the activity. (d) The shedding of ADAM17 substrates includes HB-EGF and TGF-*α* in a scratch assay in HaCaT cells. Data are shown as the mean ± SEM, *n* = 3. ^∗^*P* < 0.05 and ^∗∗^*P* < 0.01 vs the normoxia group.

To investigate whether hypoxia could alter ADAM17 expression and activity in keratinocytes, HaCaT cells were exposed to hypoxia (1% O_2_) and normoxia (21% O_2_) for the indicated time. Western blot results found that the mature protein of ADAM17 was increased significantly under hypoxia (Figures 2(a) and 2(b)). The enzyme activity of ADAM17 was assessed using a TACE protease activity kit. The results showed that hypoxia caused significant increases of ADAM17 sheddase activity (Figure 2(c)). TGF-*α* and HB-EGF are the basic substrates of ADAM17, which are mainly catalyzed by ADAM17 and are one of the important indicators for the activity of ADAM17 [26]. Therefore, we examined the shedding of ADAM17's substrates in the EGF growth factor family, including HB-EGF and TGF-*α*, when cells were cultured in hypoxic condition. The results showed that the substrate release was time dependent. After 12 h of hypoxia treatment, shedding of TGF-*α* was significantly higher in the hypoxic group than in the normoxic group. At 24 h of hypoxic condition, shedding of TGF-*α* and HB-EGF showed a sharp increase that was significantly higher than that of normoxic condition (Figure 2(d)). These results indicate that the expression and activity of ADAM17 in keratinocytes were upregulated under hypoxic conditions.

### 3.3. ADAM17 Involvement in Hypoxic-Regulated Keratinocyte Migration

**Figure 3:**
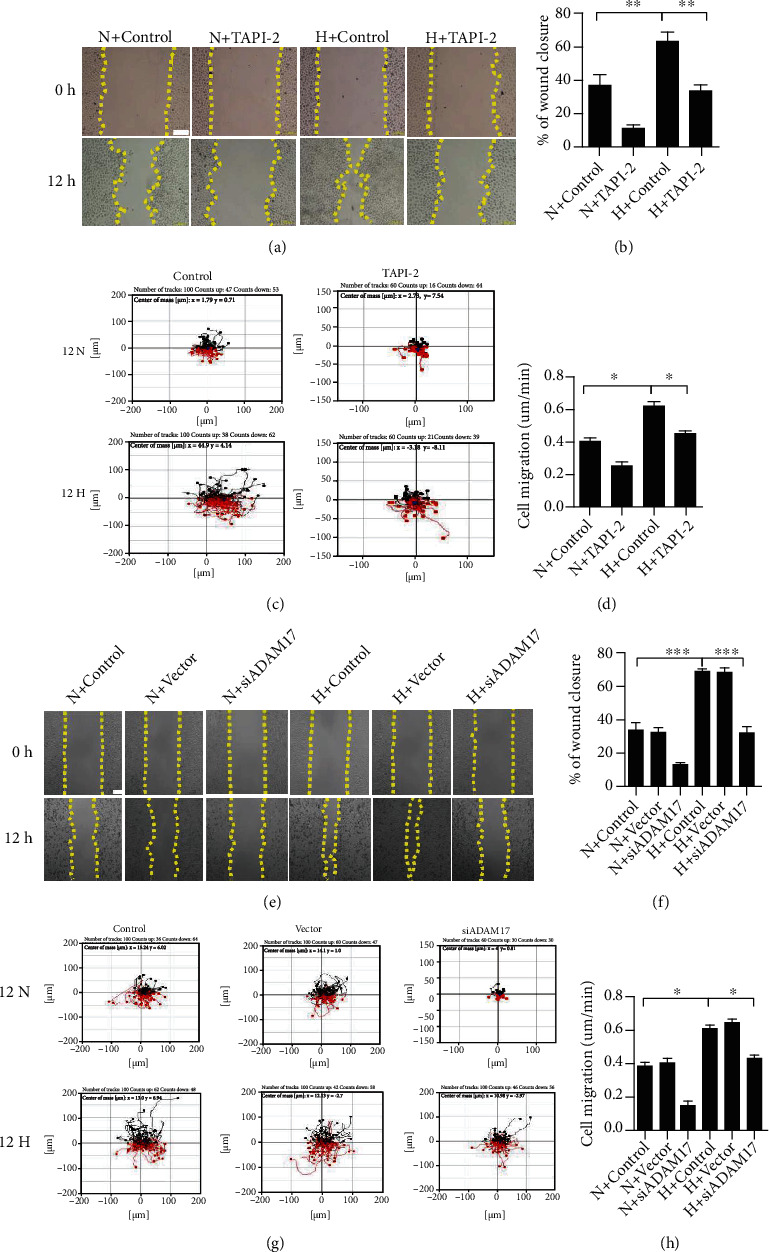
ADAM17 involvement in hypoxic-regulated keratinocyte migration. (a, e) The ADAM17 inhibitor TAPI-2 or si-ADAM17 effect on the wound closure in keratinocytes under hypoxic condition. (b, f) Quantification analysis of wound healing assays using ImageJ software. (c, g) The effect of ADAM17 inhibitor TAPI-2 or si-ADAM17 on cell motility trajectories in keratinocytes under hypoxic condition. (d, h) Statistical value of cell trajectory speed. Data were from at least 3 independent experiments and shown as the mean ± SEM. ^∗^*P* < 0.05, ^∗∗^*P* < 0.01, and ^∗∗∗^*P* < 0.001 vs the control group. Bar = 100 *μ* m.

Notably, the ranges of cell migration and motility speeds were enhanced in hypoxic condition. To investigate the role of ADAM17 in hypoxia-stimulated keratinocyte migration, we assessed the keratinocyte migration with the ADAM17 inhibitor TAPI-2 and siADAM17 as siRNA-mediated knockdown of ADAM17, using cell scratch wound assay and cell motility assay. The results of scratch wound healing assay showed that TAPI-2 treatment delayed the healing of keratinocyte monolayer wounds under hypoxia (Figure 3(a)). After TAPI-2 pretreatment, the area of wound closure was reduced by 47% in keratinocytes under hypoxic condition (Figure 3(b)). Meanwhile, compared with control, the ranges of cell migration were suppressed and the trajectory speed of keratinocytes was decreased by 27.4% in TAPI-2-pretreated keratinocytes under hypoxia (Figures 3(c) and 3(d); Supplemental Movie S2). These results suggest that ADAM17 participates in hypoxia-regulated cell movement. Furthermore, the improvement in keratinocyte migration by hypoxia treatment was abolished by siADAM17 transfection (Figures 3(e)–3(h)). After si-ADAM17 transfection, compared with that of control, the area of wound closure was reduced by 54% under hypoxic condition (Figure 3(f)). Cell motility assay showed that the trajectory speed of keratinocytes was decreased by 32.8% in siADAM17-pretreated keratinocytes under hypoxia (Figures 3(g) and 3(h); Supplemental Movie S3). These findings demonstrate that ADAM17 plays an important role in hypoxia-induced keratinocyte migration.

### 3.4. The P38/MAPK Pathway Is Involved in Hypoxia-Activated ADAM17

**Figure 4:**
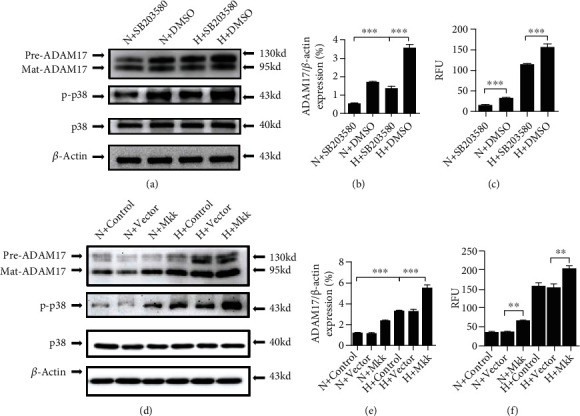
The p38/MAPK pathway is involved in hypoxia-activated ADAM17. (a) Representative Western blot showing the effect of SB203580 on p38/MAPK signal. (b) Quantification of Western blots. (c) TACE activity assay kit measurement of the effect of SB203580 on ADAM17 enzyme activity in HaCaT cells under hypoxic condition. (d) Representative Western blot showing the effect of MKK6 on p38/MAPK signal. (e) Quantification of Western blots. (f) TACE activity assay kit measurement of the effect of MKK6 on ADAM17 enzyme activity in HaCaT cells under hypoxic condition. Data are shown as the mean ± SEM, *n* = 3. ^∗∗^*P* < 0.01 vs the vector group and ^∗∗∗^*P* < 0.001 vs the control group.

To assess the role of the p38/MAPK pathway in ADAM17-mediated keratinocyte migration under hypoxia, we used a selective inhibitor of p38/MAPK and SB203580, which inhibits the catalytic activity of p38 MAPK by competitive binding in the ATP pocket and has been shown to inhibit p38 MAPK in vivo. The results showed that hypoxia induced ADAM17 expression and p38/MAPK activation as expected; this was prevented by the addition of SB203580 (Figures 4(a)–4(c)). Compared with control, SB203580 also attenuated ADAM17 activity in keratinocytes under hypoxic condition (Figure 4(c)). To further confirm a potential role of p38/MAPK in ADAM17, HaCaT cells were transfected with MKK6 (Glu) recombinant adenovirus (Figure 4(d)). MKK6 is known to induce p38/MAPK activation by phosphorylating p38/MAPK on Thr-180 and Tyr-182 [27]. As shown in Figures 4(d)–4(f), MKK6 (Glu) overexpression significantly increased the expression and activity of ADAM17 in keratinocytes. The requirement of p38/MAPK for ADAM17 activation under hypoxia raised the possibility that p38/MAPK is a critical signaling pathway in hypoxia-induced upregulation of ADAM17.

### 3.5. Hypoxia Induced Keratinocyte Migration via the P38/MAPK Pathway

**Figure 5:**
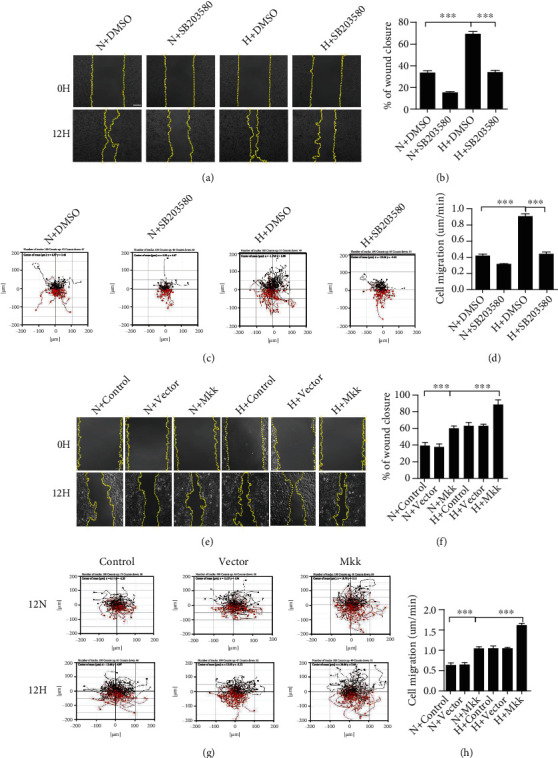
Hypoxia-induced keratinocyte migration via the p38/MAPK pathway. (a, e) The effect of SB203580 on the wound closure in keratinocytes under hypoxic condition. (b, f) Quantification analysis of wound healing assays using ImageJ software. (c, g) The effect of SB203580 on cell motility trajectories in keratinocytes under hypoxic condition. (d, h) Statistical value of cell trajectory speed. Data were from at least 3 independent experiments and shown as the mean ± SEM. ^∗∗∗^*P* < 0.001 vs the control group. Bar = 100 *μ*m.

To obtain further insight into the role of p38/MAPK signaling in hypoxia-induced keratinocyte migration, we performed scratch wound healing assays with HaCaT cells cultured in the presence of SB203580 for 12 hours. Scratch wound healing was notably inhibited by SB203580 treatment under hypoxia (Figures 5(a)–5(b)). In addition, we detected keratinocyte motility treated with SB203580. The results showed a significant decrease in the range of cell movement and the speeds of cell migration (Figures 5(c) and 5(d)). Importantly, keratinocyte migration capacity was strongly increased by transfected MKK6 (Glu) to activate p38/MAPK signaling. The scratch wound healing assay showed that MKK6 (Glu) overexpression remarkably promoted HaCaT cell migration (Figures 5(e) and 5(f)). An *in vitro* cell motility assay was then found to have a notable increase in the range of keratinocyte movement and the velocity of cell migration by transfected MKK6 (Glu) (Figures 5(g) and 5(h); Supplemental Movie S4). These results revealed an important role for p38/MAPK in hypoxia-induced keratinocyte migration. Taken together, our findings therefore proposed that the hypoxia-induced keratinocyte migration is mediated by ADAM17 through a p38/MAPK-dependent pathway.

## 4. Discussion

As a key therapeutic target in human disease, hypoxia plays a critical role in angiogenesis, heart regeneration, cell migration, and cancer invasion [28]; its underlying molecular mechanisms however are largely unknown. With gain- and loss-of-function studies together with pharmacological inhibitors, we here showed that hypoxia promoted ADAM17 expression and activity, which played a pivotal role in hypoxia-induced keratinocyte migration. Moreover, we speculated that the hypoxia-upregulated ADAM17 was dependent on p38/MAPK signaling. Our study shed new light on the molecular mechanisms of the well-established function of hypoxia in skin homoeostasis and wound healing.

Hypoxic stress has been identified as an important regulator of cell homeostasis. Studies have shown that hypoxia plays critical roles in epithelial cell migration and is associated with almost every step of wound healing, including cell proliferation, collagen synthesis, reepithelialization, and defense against bacteria. After an acute injury, gene expression and growth factor synthesis are activated by hypoxia, which leads to tissue repair/angiogenesis [29]. Indeed, in the early proliferative phase, hypoxia can be detected in reepithelializing sheets by pimonidazole adduct staining; such adducts can bind irreversibly to thiol groups in amino acids when PO_2_ < 10 mmHg (1.3% O_2_) [29]. To assess whether hypoxia induces keratinocyte migration, we utilized cell scratch wound healing assay and cell motility assay. The results of cell scratch wounding assay showed that the migration of keratinocytes was enhanced after culture in hypoxic condition (1% O_2_) compared to that in normoxic condition (21% O_2_) (Figures 1(a) and 1(b)). Transwell assays could be conducted in the future to further evaluate cell migration. The mechanisms underlying the hypoxia-induced regulation of keratinocyte migration are not well understood. In our previous studies, hypoxia can promote the migration of epidermal cells through signaling pathways such as CD9 [8, 30]. The results show that hypoxia downregulates CD9 by activating the p38/MAPK pathway, which promotes keratinocyte migration. Nevertheless, tetraspanin CD9 is not generally believed to have ligands or to function as a cell surface receptor; rather, it is thought to associate with other transmembrane molecules, thereby mediating keratinocyte migration. Our latest research demonstrated that the CD9/ADAM17 axis played a key role in keratinocyte migration via activation of the EGFR/ERK signaling pathway [31]. Previous studies have found that the inhibition of ADAM17 activity reduces hypoxia-induced brain tumor cell invasion [32]. In addition, the stimulation of ADAM17 activity promotes cell migration and the TGF-*α*/EGFR pathway in invasive hypoxia prostate cancer cells [33]. The above results raised the possibility that ADAM17 is a critical intermediate in hypoxia-regulated cell migration.

Growing evidence has demonstrated that the ADAM family could be a potential target in wound healing. The ADAM family consists of 22 members; importantly, only ADAM17 is thought to be a key regulator in the repair of skin [34]. After acute epidermal injury, hypoxia induces a series of adaptive physiological responses, including the expression of a selected set of genes required for tissue repair/angiogenesis [31]. ADAM17 is strongly stimulated in response to hypoxia. As expected, we observed that hypoxia upregulated ADAM17 expression and activity significantly in keratinocytes (Figure 2). In various cell tapes, the sheddase activity of ADAM17 modulates several cellular processes, including cell invasion, motility, and migration [26, 35]. It was previously demonstrated that ADAM17 contributed to tumor cell migration and invasion in vitro [36]. Our results also confirmed that the promoting effects of hypoxia on keratinocyte migration could be attenuated by ADAM17 inhibitor-TAPI-2 and ADAM17 siRNA (Figure 3). These results suggest that ADAM17 plays a critical role in keratinocyte migration under hypoxic condition.

It has been shown that p38/MAPK signaling plays a promotional role in the wound healing process. An in vitro study demonstrated that a mutant in p38-*α* or p38-*β* MAPK attenuated wound healing [37]. However, whether p38/MAPK is involved in ADAM17 mediating hypoxia-induced keratinocyte migration has not been elucidated. There are numerous signals such as ROS, PKC, Src, and MAPK that could interact directly with ADAM17 [38]. Extracellular stimuli, such as p38/MAPK, can interact directly with the cytoplasmic domain of ADAMs and phosphorylate it at Thr735 to regulate its proteolytic activity and substrate recognition [39]. In addition, the activation of ADAM17 by p38/MAPK resulted in the release of TGF-*α* family ligands, which activate EGF receptor signaling [22]. Thus, we hypothesized that p38/MAPK plays a role in hypoxia-induced ADAM17 expression in keratinocytes. In this work, we also observed that the p38/MAPK phosphorylation was enhanced by hypoxia treatment. The hypoxia-upregulated ADAM17 expression and activation could be suppressed by the p38/MAPK inhibitor or promoted by MKK6, an upstream kinase of p38/MAPK in keratinocytes. Therefore, hypoxia induced p38/MAPK activation, which in turn was recruited as an ADAM17 promoter and ultimately induced ADAM17 expression and activity. These results suggest a novel signaling mechanism in hypoxia-induced keratinocyte migration that involves a p38/MAPK-ADAM17 axis.

In conclusion, our findings demonstrated that ADAM17 expression and activity were promoted by hypoxia through a p38/MAPK signaling, which account for the induced migration of keratinocytes under hypoxic condition. Our study shed new light on the regulatory mechanisms in keratinocyte migration that may help develop a therapeutic strategies to facilitate wound healing in vivo, where guided cell migration in hypoxic microenvironment is needed.

## Data Availability

The data used to support the findings of this study are available upon request.

## Supplementary Materials

Supplementary Materials HaCaT cells were transfected with Lipofectamine 2000 (Invitrogen) following the manufacturer's instructions. Small interfering RNA was designed to silence ADAM17 (sequence information of three sites). Detailed information is shown in Supplemental Table 1: movie S1 video of cell motility assay to observe the effect of hypoxia on the migration ability of single cell in HaCaT cells. Movie S2 video of cell motility assay to observe the effect of ADAM17 inhibitor TAPI-2 on the migration ability of single cell in HaCaT cells. Movie S3 video of cell motility assay to observe the effect of si-ADAM17 on the migration ability of single cell in HaCaT cells. Movie S4 video of cell motility assay to observe the effect of MKK6 overexpression on the migration ability of single cell in HaCaT cells.
