# Supplementary material for: ADAM17 Mediates Hypoxia-Induced Keratinocyte Migration via the p38/MAPK Pathway
Source: Biomed Res Int. 2021 Oct 28;2021:8328216. doi: 10.1155/2021/8328216 (PMC8568513; doi:10.1155/2021/8328216)
Supplement: Supplementary Materials — HaCaT cells were transfected with Lipofectamine 2000 (Invitrogen) following the manufacturer's instructions. Small interfering RNA was designed to silence ADAM17 (sequence information of three sites). Detailed information is shown in Supplemental Table 1: movie S1 video of cell motility assay to observe the effect of hypoxia on the migration ability of single cell in HaCaT cells. Movie S2 video of cell motility assay to observe the effect of ADAM17 inhibitor TAPI-2 on the migration ability of single cell in HaCaT cells. Movie S3 video of cell motility assay to observe the effect of si-ADAM17 on the migration ability of single cell in HaCaT cells. Movie S4 video of cell motility assay to observe the effect of MKK6 overexpression on the migration ability of single cell in HaCaT cells. [file 8328216.f1.zip › Supplemental table 1.docx]

Table I .The ADAM17 target sequences

| **Specific** | **sense** | **antisense** |
| --- | --- | --- |
| 842 | 5’-CCAUGAAGAACACGUGU | 5’-UUACACGUGUUCUUCAUGGT |
| 1432 | 5'-CCGGAUGGUCUAGCAGA | 5'-AUUCUGCUAGACCAUCCGGT |
| 1658 | 5'-GCAUCAUGUAUCUGAAC | 5'-UUGUUCAGAUACAUGAUGCT |
| NC | 5’-UUCUCCGAGCGUGUCAC | 5’-ACGUGACAAGUUAGGAGAAT |

NC: Negative Control

HaCaT cells were transfected with Lipofectamine 2000 (Invitrogen) following the manufacturer's instructions.
